# Responses of the growth, photosynthetic characteristics, endogenous hormones and antioxidant activity of *Carpinus betulus* L. seedlings to different light intensities

**DOI:** 10.3389/fpls.2022.1055984

**Published:** 2022-12-01

**Authors:** Qi Zhou, Feng Zhao, Huihui Zhang, Zunling Zhu

**Affiliations:** ^1^ School of Environment and Ecology, Jiangsu Open University, Nanjing, China; ^2^ Co-Innovation Center for Sustainable Forestry in Southern China, Nanjing Forestry University, Nanjing, China; ^3^ School of Engineering and Architecture, Jiangsu Open University, Nanjing, China; ^4^ College of Landscape Architecture, Nanjing Forestry University, Nanjing, China

**Keywords:** *Carpinus betulus*, light intensity, morphological indexes, physiology, photosynthetic responses, endogenous hormones, antioxidant activity

## Abstract

Light is an important ecological factor that affects plant growth, survival and distribution. *Carpinus betulus* L. is native to central Europe and is used as an ornamental plant with strong adaptability. It is an important tree species for landscaping and timber use. What’s more, the antioxidant- and anticancer-related properties of *C. betulus* leaf extracts are remarkable, that make it a possible raw material for medicine. Light intensity is an important environmental factor affecting the growth and physicochemical changes of *C. betulus*, but the mechanism of its effect on this species still remains unknown. In this study, the growth, photosynthetic characteristics, endogenous hormones and antioxidant activity responses of *C. betulus* seedlings to four light intensity gradients (T0: normal light; T1: 75%; T2: 50%; T3: 25% of normal light) were studied after 60 days of shading treatments. The results showed a significant effect of low light intensity on the values of the growth and physiological parameters of *C. betulus*. The low light intensity caused the inhibition of plant biomass accumulation and the degradation of photosynthetic capacity and stomatal behavior and aggravated the cell membrane lipid peroxidation. However, the plant height growth, leaf area, specific leaf area, photosynthetic pigment content, and contents of GA_3_ and ABA of *C. betulus* increased with decreasing light intensity. We found that *C. betulus* can tolerate mild (T1) and moderate (T2) shading stress by developing photoprotective mechanisms and maintaining relatively high concentrations of organic osmolytes and high antioxidant enzyme activities (superoxide dismutase, peroxidase, catalase and ascorbate peroxidase), but the ability of *C. betulus* to synthesize osmotic substances and enzymatic antioxidants was impaired under severe shading conditions (T3). Our results suggest that *C. betulus* can make effective use of low light resources by adjusting its morphology, material distribution, photosynthetic rate and antioxidant enzyme system in suitable low-light environments (50%~75% of normal light); however, light intensity levels that are too low (25% of normal light) will have adverse effects on plant growth. The results of this study provide not only new insights into the response mechanisms of *C. betulus* to light intensity but also a scientific basis for the cultivation and application of *C. betulus* in China.

## Introduction

Light is the material basis with which all plants in nature carry out various life-sustaining activities (Kami et al., 2010). Light is a primary determinant of the geographical distribution and growth of plants through light intensity, light quality, photoperiod and other factors (Jason et al., 2018). As one of these important factors, light intensity has an important impact on plant growth, morphology, physiological metabolism, signal transduction and photosynthetic physiology (Fan et al., 2013; Lu et al., 2018). The relationship between plants and the light environment has always been a hot topic in ecology.

When the light intensity in the environment changes, the growth state of plants will inevitably change to a certain extent, and this change is mainly caused by the influence of the active light capture ability of the plant and the ability to synthesize photosynthates (Chen et al., 2014). Studies have found that in a weak light environment, the leaf area, total biomass, specific leaf area, and leaf area ratio of plants will increase, thus making leaves larger and thinner, increasing the area of light interception, improving the utilization efficiency of limited light resources, increasing stomatal conductance, and maintaining a high photosynthetic rate to ensure normal plant functions in low-light environments (Guo et al., 2019; Pan et al., 2020). Moreover, the absorption and utilization of light energy can be enhanced in plants growing in low-light conditions through increases in chlorophyll content, apparent quantum efficiency, actual photochemical quantum yield of PSII (Φ_PSII_) and electron transfer rate (ETR) (Guenni et al., 2018; Shi et al., 2018) and reductions in energy waste by reducing the light compensation point, dark respiration rate, photorespiration rate and heat dissipation ratio (Cai et al., 2017; Ghorbanzadeh et al., 2020). However, in a strong light environment, plants can reduce the capture of excess light and the damage caused to plants by increasing leaf thickness, reducing leaf area, reducing chlorophyll content, and reducing the transpiration of leaf water by closing stomata (Mokany et al., 2006).

Through the long evolutionary process, plants have formed a series of protective mechanisms that enable them to adapt to the complex and dynamic light environment. Studies have found that when plants grow in an unsuitable light environment, osmotic regulatory substances such as soluble sugar (SS), soluble protein (SP) and proline (Pro) can maintain the balance of cell osmotic potential and reduce the damage to the cell membrane caused by osmotic stress through changes in content (Yin and Shen, 2016). Antioxidant systems also play an important role in plants coping with unfavorable light conditions, resulting in the accumulation of intracellular reactive oxygen species (ROS) when plants are exposed to environmental stress; thus, the activities of protective enzymes such as superoxide dismutase (SOD), catalase (POD) and peroxygenase (CAT) and the content of nonenzymatic antioxidants increase (Liu Y. et al., 2018; Zushi et al., 2020), and the contents of active oxygen species such as O^·-2^, H_2_O_2_, OH^-^ and malondialdehyde (MDA) decrease. These changes can reduce peroxidation damage to the cell membrane and improve the adaptability of plants to unfavorable light environments (Ellouzi et al., 2011). Plants tolerate shade stress in a variety of ways (Israeli et al., 1995; Koike, 2010; Modrzyński et al., 2015). Some studies have found that moderate shade can benefit plants not only by preventing them from being burned by light but also by allowing them to meet their light requirements for growth (Petritan et al., 2007). Therefore, it is important to study how plants tolerate shade stress to more effectively establish stable, diverse and stratified planting structures when constructing landscape gardens.

*Carpinus betulus* L. is a deciduous tree belonging to the Betulaceae family with beautiful foliage and golden leaves in autumn. It plays an important role in forest communities in temperate regions of Europe and Asia Minor (Sikkema et al., 2016) and has been introduced and cultivated in many places in China with good ecological adaptability (Shi et al., 2017). In landscapes, *C. betulus* is often planted in the form of street trees or hedgerows and is well-known in private and public green areas for its autumn leaves. It is a suitable species for both ornamental and urban habitats and has been used for biological monitoring of particulate matter (Prinz and Finkeldey, 2015). Additional studies have shown that the antioxidant- and anticancer-related properties of *C. betulus* leaf extracts are exceptional (Cieckiewicz et al., 2012; Hofmann et al., 2016) and that compared to other common coniferous and deciduous trees, these extracts contain very high amounts of total phenols and phenolic acids (Kuiters and Sarink, 1986). All these results indicate that *C. betulus* leaves are a promising renewable resource for the biorefinery and utilization of its antioxidant extractives in the future for healthcare and medical products. The high ornamental and economic value of *C. betulus* makes it extremely popular globally. In recent years, *C. betulus* has been widely used in China, and its adaptability is good. Recent studies on *C. betulus* have mostly concentrated on breeding (Chalupa, 1990), seed biology (Zhu et al., 2013), heat resistance (Shi and Zhu, 2015), drought and salt tolerance (Stojnić et al., 2016; Zhou et al., 2018). At present, with the rapid development of urban construction in China, the urban shade space is becoming larger, air pollution and smog get more severe in big cities (Xue et al., 2022). It is of great significance to select the plant resources with high ornamental value and shade tolerance to carry out scientific and effective greening of the shade space and give play to the best ecological environmental benefits of green space. Light has a great influence on the growth of *C. betulus.* However, information on the response of *C. betulus* to different light intensities is scarce.

Therefore, the main objective of this study is to comprehensively assess the influence of shade stress on *C. betulus* and to determine the mechanisms of adaptation employed by this plant to tolerate low-light stress. We hypothesized that the growth, biomass accumulation and leaf gas exchange rate of *C. betulus* seedlings would decrease under the effects of light stress. We examined the growth and physicochemical changes in seedlings grown under different degrees of shading created through artificial shading treatment, explored the adaptability of *C. betulus* seedlings to different light conditions, and sought to determine the most suitable light conditions to promote the growth and development of *C. betulus* seedlings. The aim of this study was to provide scientific data and findings related to the light management of *C. betulus* and to make better use of *Carpinus* species in landscaping and industrial production.

## Materials and methods

### Plant materials and light treatments

The tests were carried out in the Landscape Experimental Teaching Center of Nanjing Forestry University in May, Nanjing (33° 04’ N, 118° 47’ E), Jiangsu Province. The area is characterized by a warm and humid subtropical monsoon climate with an annual rainfall of 1200 mm. The average annual temperature is 15.7°C, the maximum temperature is 40.7°C, and the minimum temperature is -14°C. Two-year-old *C. betulus* seedlings were obtained from Nanjing Forestry University. On April 2016, we selected well-grown seedlings with a similar mean ground diameter (0.7-0.8 cm) and seedling height (50-60 cm) and transplanted them into pots (20 cm diameter × 22 cm height). The potting soil was a soil:peat:vermiculite:pearlite (1:1:1:1, v:v:v:v, pH 6.50) mixture. The substrate was imported with an organic matter content of 75.5 g/kg, total P content of 2.80 g/kg, total N content of 73.5 g/kg, and total K content of 9.0 g/kg. The content of soil nutrients was determined by a TFW-VI soil nutrient and moisture tester (TFW-VI, Wuhan, China). The pots were then placed in a glasshouse at a temperature of 25 ± 2.0°C and relative humidity of 70 ± 5%, with standardized fertilizer and water management.

Light treatments were conducted in June 2016 outside of the greenhouse. Four types of light treatments were administered as follows: no shading net (T0, CK, 100% normal light), 25% shading net (T1, 75% normal light), 50% shading net (T2, 50% normal light) and 75% shading net (T3, 25% normal light). These four types of shading net are common in the market; the shading net was 1.8 m away from the ground to simulate natural shading, and a TES-1334A was used to determine the light intensity. Each treatment had 3 replicates with 15 plants in each replicate and one seedling per basin (i.e., 45 seedlings per treatment). All the seedlings were watered to field capacity twice a week during the summer months. Weeding was carried out when needed. After 60 days of growth, measurements were carried out, and samples were collected for various physiological analyses, with three replicates for the measured parameters. When sampling, we chose mature new leaves pointing in the same direction from the upper part of *C. betulus*. Fresh leaves were collected from plants, immediately frozen in liquid nitrogen and stored at -80°C. The leaves were used for the determination of physiological and biochemical indexes.

### Plant growth measurement

Before the shading stress treatment, seedling height (H_0_) and diameter (D_0_) were determined from three seedlings in each group. After 60 days of treatment, the height (H_1_) and diameter (D_1_) were determined again. Plant height increment was calculated as H_1_ − H_0_, and ground diameter increment was calculated as D_1_ − D_0_. Fully expanded fresh leaves (the third or fourth leaf from the top of the shoots) were measured before biomass harvest using a portable leaf area meter (LI-3000C, LI-COR, Lincoln, Nebraska, USA). Then, the plants were harvested, the samples were separated into roots, stems and leaves, and the taproot length (roots were washed with distilled water, blotted dry on filter paper, measured the taproot length with a ruler) was calculated. The samples were dried at 80°C in an oven for 48 h, and the dry weights of the samples were recorded. The specific leaf area (SLA) was calculated: SLA (cm^2^/g) = leaf area (cm^2^)/leaf dry weight (g). Portions of these samples were frozen in liquid nitrogen and stored at −80°C.

### Scanning electron microscopy of leaf stomatal and section characteristics

The third or fourth fresh leaf from the top of *C. Betulus* seedlings was collected on the final day of the shading treatment from the three seedlings in each group. The cleaned leaves were cut into small pieces (approximately 5 × 5 mm) with a sharp blade and then placed in formalin acetic acid (FAA). After dehydration in a graded ethanol series, penetration with isoamyl acetate aldehyde, and drying in a critical point drying apparatus (K850, Emitech, London, UK), the samples were mounted on stubs and coated with gold using an ion sputtering apparatus (E1010, Hitachi, Tokyo, Japan). Then, a scanning electron microscope (SEM, Quanta 200, FEI, Hillsborough, Oregon, USA) was used to observe the blade surface and cross-section of the leaves, and images were taken using the same instrument. The stomatal density (number/mm^2^) and stomatal size (length and width) were evaluated following the method reported by Camposeo et al. (2011). The number of open stomata, leaf thickness (μm) and palisade tissue thickness (μm) for each sample were measured under a photomicroscope system with a computer attachment. Stomatal aperture (μm) was measured by the following method: three complete and clear stomata in each field were randomly selected, and stomatal opening (the maximum value of pores formed between guard cells) was measured.

### Leaf gas exchanges and chlorophyll fluorescence parameters

Photosynthetic parameters were determined in the third to fourth fully expanded fresh leaves of each plant from 8:00~11:00 a.m on the final day of the different light treatments using a portable photosynthetic system (Ciras-2, Shanghai, China). The parameters recorded included the net photosynthetic rate (Pn), transpiration rate (Tr), stomatal conductance (Gs), and intercellular CO_2_ concentration (Ci). Chlorophyll fluorescence parameters were determined by the fluorescence leaf chamber of the Ciras-2 photosynthetic system on the same day. The potential activity of PSII (Fv/Fo), maximum photochemical efficiency of photosystem II (Fv/Fm), photochemical quantum efficiency (Φ_PSII_), photochemical quenching parameter (qP), nonphotochemical quenching parameter (NPQ), and electron transfer rate (ETR) were measured on fully dark-adapted leaves. Three seedlings were randomly selected for each treatment, and the parameter measurements were performed on three leaves per plant.

### Determination of photosynthetic pigment contents

The chlorophyll a (Chl a), chlorophyll b (Chl b), and total carotenoid (Car) contents of the fresh leaves in the different light treatments were measured with the spectrophotometric method (Porra et al., 1989). Chlorophyll from 4~5 pieces of fresh leaves was extracted with a mixture of acetone and 95% ethanol (1:1, v:v), and the absorbance of the samples was measured at wavelengths of 645 nm and 663 nm by ultraviolet‐visible spectrophotometry (Lambda25, PerkinElmer, Waltham, Massachusetts, USA). Then, the photosynthetic pigment contents (mg/g) were calculated with three replicates.

### Determination of lipid peroxidation

Lipid peroxidation of fresh leaves under the different light conditions was assessed by malondialdehyde (MDA) content and relative electrolytic conductivity (REC) with three replicates. The MDA content was measured following the method of Hodges et al. (1999). The relative electrolytic conductivity (REC) was determined according to the method of Dionisio-Sese and Tobita (1998).

### Organic osmolytes

Three main types of organic osmolytes, including soluble sugars, soluble proteins, and proline, were analyzed. The soluble sugar content of leaves under the different light conditions was measured by the anthrone colorimetric method according to the method reported by Magné et al. (2006). The content of soluble protein was measured by Coomassie brilliant blue G-250 staining (Bradford, 1976). Proline was extracted from the leaves of *C. betulus* and determined by the method described by Abrahám et al. (2015).

### Endogenous hormones

The contents of auxin (IAA), abscisic acid (ABA) and gibberellin (GA_3_) in leaves of *C. betulus* under different light treatments were detected by HPLC‐MS/MS by the method reported by Ma et al. (2008). The mobile phase contained 0.1% formic acid and was eluted by gradient reversed-phase HPLC. The pH was adjusted to 3.2 with methanol-modified triethylamine (TEA), and the solute was detected at a wavelength of 265 nm.

### Antioxidant enzymes

0.3 g frozen leaves were ground at 4°C in a mortar in 6 mL of a pH 7.8 phosphate buffer solution. The homogenate was centrifuged at 10,000 rpm at 4°C for 20 min. The supernatant was collected as a leaf crude enzyme extract for enzyme measurements and stored at 4°C. The activities of superoxide dismutase (SOD), peroxidase (POD), catalase (CAT) and ascorbate peroxidase (APX) were determined in the leaf enzyme extracts of *C. betulus*. SOD activity was analyzed following Beyer and Fridovich (1987). POD activity was estimated according to the method reported by Civello et al. (1995). CAT activity was measured following Díaz-Vivancos et al. (2008). APX activity was measured using the method described by Nakano and Asada (1981).

### Statistical analysis

Data were analyzed by calculating the means and standard deviation (SD) using one-way ANOVA, and the means were separated with Duncan’s multiple range test at the 5% probability level using SPSS statistical package version 22.0 (IBM Corp, Amonk, New York, USA). All tables and graphs were made using MS Excel 2019.

## Results

### Effects of light intensity on plant growth

**Figure 1:**
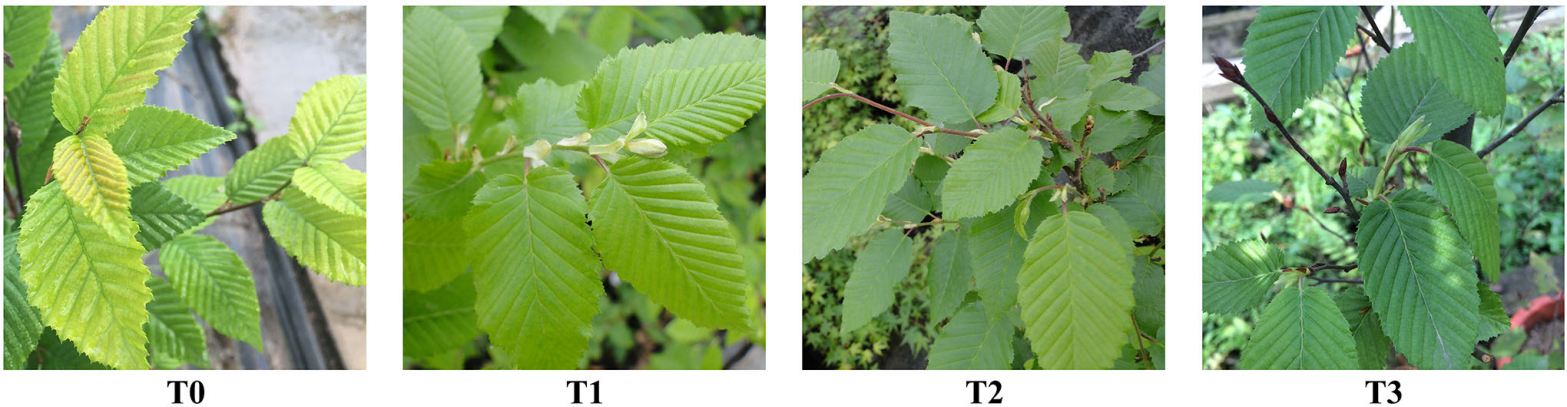
The growth of the leaves of *C. betulus* seedlings under different light intensity (T0~T3) after 60 days.

Plant growth exhibits a certain adaptability to changes in light intensity. The *C. betulus* seedlings could maintain basically normal growth, and the survival rate was 100% throughout the study. However, there were significant differences in plant growth under different shading treatments (T1~T3), the leaf color of *C. betulus* seedlings gradually darkens compared with the CK as the degree of shading increased (**Figure 1**). As shown in **Table 1**, the plant height growth, leaf area, and SLA of *C. betulus* increased with decreasing light intensity, and those indicators were significantly higher (*P* < 0.05) in the plants grown under the shaded conditions (T1~T3) than in the control plants (T0). However, the ground diameter increment, taproot length, root biomass, stem biomass, leaf biomass, root:shoot ratio, and total biomass were inhibited by shade stress. The taproot length and total biomass of the treatment group were significantly lower than those of the control group.

**Table 1:** Effects of various light intensities on plant height increment (cm), ground diameter increment (cm), taproot length (cm), leaf area (cm^2^), specific leaf area (SLA, cm^2^/g), roots, stems and leaf biomass (g), root:shoot ratio (R:S) and total biomass (g) after 60 days of treatment.

Treatment	Plant height increment (cm)	Ground diameter increment (cm)	Taproot length (cm)	Leaf area (cm^2^)	Specific leaf area (SLA,cm^2^/g)
**T0**	5.26 ± 0.32d	0.16 ± 0.011a	20.86 ± 1.82a	12.58 ± 1.67c	299.52 ± 8.16d
**T1**	6.52 ± 0.58c	0.12 ± 0.019ab	18.15 ± 1.25b	13.98 ± 1.26b	367.89 ± 12.08c
**T2**	7.82 ± 1.48b	0.10 ± 0.012b	16.91 ± 0.83c	14.22 ± 1.58ab	406.28 ± 15.15b
**T3**	8.54 ± 1.76a	0.08 ± 0.008c	14.45 ± 1.06d	14.98 ± 1.42a	447.27 ± 16.32a
**Treatment**	**Root biomass (g)**	**Stem biomass (g)**	**Leaf biomass (g)**	**Root:shoot ratio (R:S)**	**Total biomass (g)**
**T0**	4.25 ± 0.24a	3.38 ± 0.18a	3.46 ± 0.17a	0.62 ± 0.021a	11.09 ± 0.32a
**T1**	3.73 ± 0.32ab	3.06 ± 0.21ab	3.15 ± 0.21ab	0.60 ± 0.015ab	9.94 ± 0.45b
**T2**	3.32 ± 0.19bc	2.89 ± 0.15b	2.87 ± 0.25b	0.58 ± 0.018b	9.08 ± 0.22bc
**T3**	2.92 ± 0.12c	2.58 ± 0.13c	2.63 ± 0.16c	0.56 ± 0.017b	8.13 ± 0.13c

Data in the table are the means ± SDs (n = 3); different lowercase letters in each column indicate significant differences among treatments (P < 0.05).

### Effects of light intensity on leaf stomatal and section characteristics

**Figure 2:**
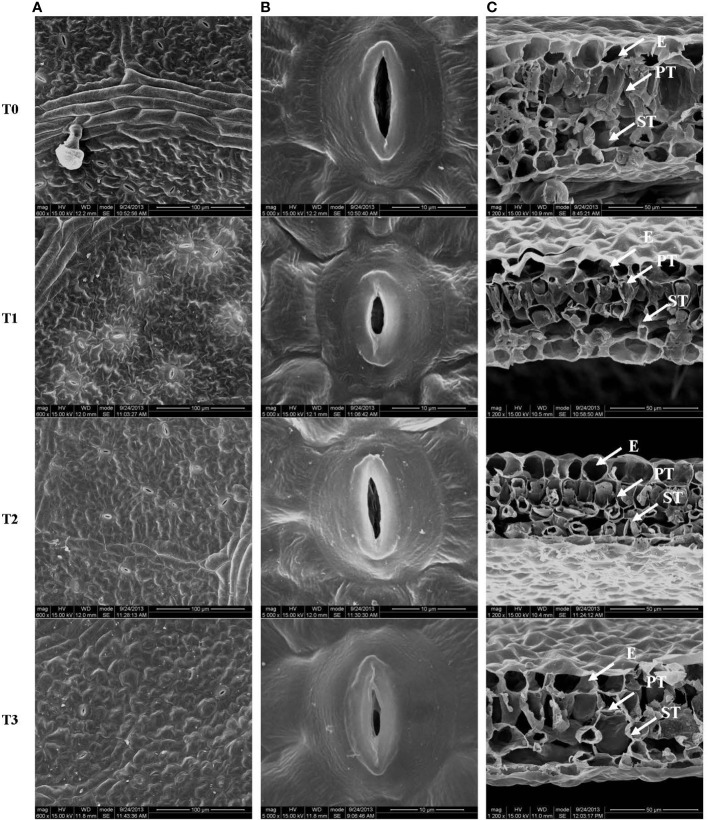
Stomatal structure on the leaf surface **(A, B)** and leaf cross-section **(C)** from *C. betulus* leaves under different light intensity treatments (T0~T3). E, epidermis; PT, palisade tissue; ST, spongy tissue. Scale: 100 μm **(A)**, 10 μm **(B)**, 50 μm **(C)**.

The effects of light intensity on leaf stomatal and section characteristics are shown in **Table 2** and **Figure 2**. The stomatal density and opened stomata of the *C. betulus* leaves from plants grown under the T1 treatment were not significantly different from those grown under the T0 treatment. However, with increased shading (T2~T3), except for the palisade:spongy ratio and palisade tissue thickness:leaf thickness ratio, all the other morphological indicators of leaves (**Table 2**) decreased significantly compared with those of the control group (*P* < 0.05). In the T3 treatment, the stomatal density was 73.27% of that of the control, and the stomata length, stomata width, stomatal aperture and leaf thickness were reduced to nearly half of those of the control. Moreover, only 35.58% of the open stomata was observed in the T3 group. Although the palisade tissue thickness and spongy tissue thickness of the leaves of treated plants were reduced with decreased light intensity, their ratio increased gradually. The palisade tissue thickness:leaf thickness ratio in T1 was lower than the control, but it was significant higher than the control in T2 and T3 treatments.

**Table 2:** The main effects of light intensity on leaf stomata and leaf section characteristics of *C. betulus*.

Treatment	Stomatal density (number/mm^2^)	Stomatal length (μm)	Stomatal width (μm)	Stomatal aperture (μm)	Open stomata (%)
**T0**	245.69 ± 23.46a	16.28 ± 2.24a	10.82 ± 1.33a	2.28 ± 0.31a	98.25 ± 6.08a
**T1**	238.58 ± 21.12ab	12.53 ± 1.48b	9.02 ± 1.58b	1.92 ± 0.27b	90.36 ± 4.64ab
**T2**	215.46 ± 18.28b	10.65 ± 1.82bc	7.35 ± 1.66c	1.70 ± 0.23bc	75.85 ± 5.86b
**T3**	180.01 ± 16.53c	8.07 ± 1.56c	4.56 ± 1.02d	1.31 ± 0.12c	35.58 ± 3.62c
**Treatment**	**Leaf thickness (μm)**	**Palisade tissue thickness (μm)**	**Spongy tissue thickness (μm)**	**Palisade/spongy (%)**	**Palisade tissue thickness/leaf thickness (%)**
**T0**	86.21 ± 4.88a	20.69 ± 2.32a	27.58 ± 3.06a	75.02 ± 3.06c	24.00 ± 3.68c
**T1**	77.58 ± 7.54b	17.24 ± 2.54b	20.69 ± 2.68b	83.33 ± 2.82b	22.22 ± 2.84d
**T2**	60.34 ± 5.72c	15.51 ± 1.68c	17.35 ± 1.50c	89.39 ± 3.52b	25.70 ± 2.55b
**T3**	44.83 ± 6.68d	14.10 ± 1.22c	14.82 ± 1.69d	95.14 ± 4.03a	31.45 ± 3.26a

The data in the table are the means ± SDs (n=3); different lowercase letters in each column indicate significant differences between treatments (P < 0.05). The parameters measured included stomatal density (number/mm^2^), stomatal length (μm), stomatal width (μm), stomatal aperture (μm), open stomata (%), leaf thickness (μm), palisade tissue thickness (μm), spongy tissue thickness (μm), palisade tissue thickness/spongy tissue thickness (%) and palisade tissue thickness/leaf thickness (%).

**Figure 3:**
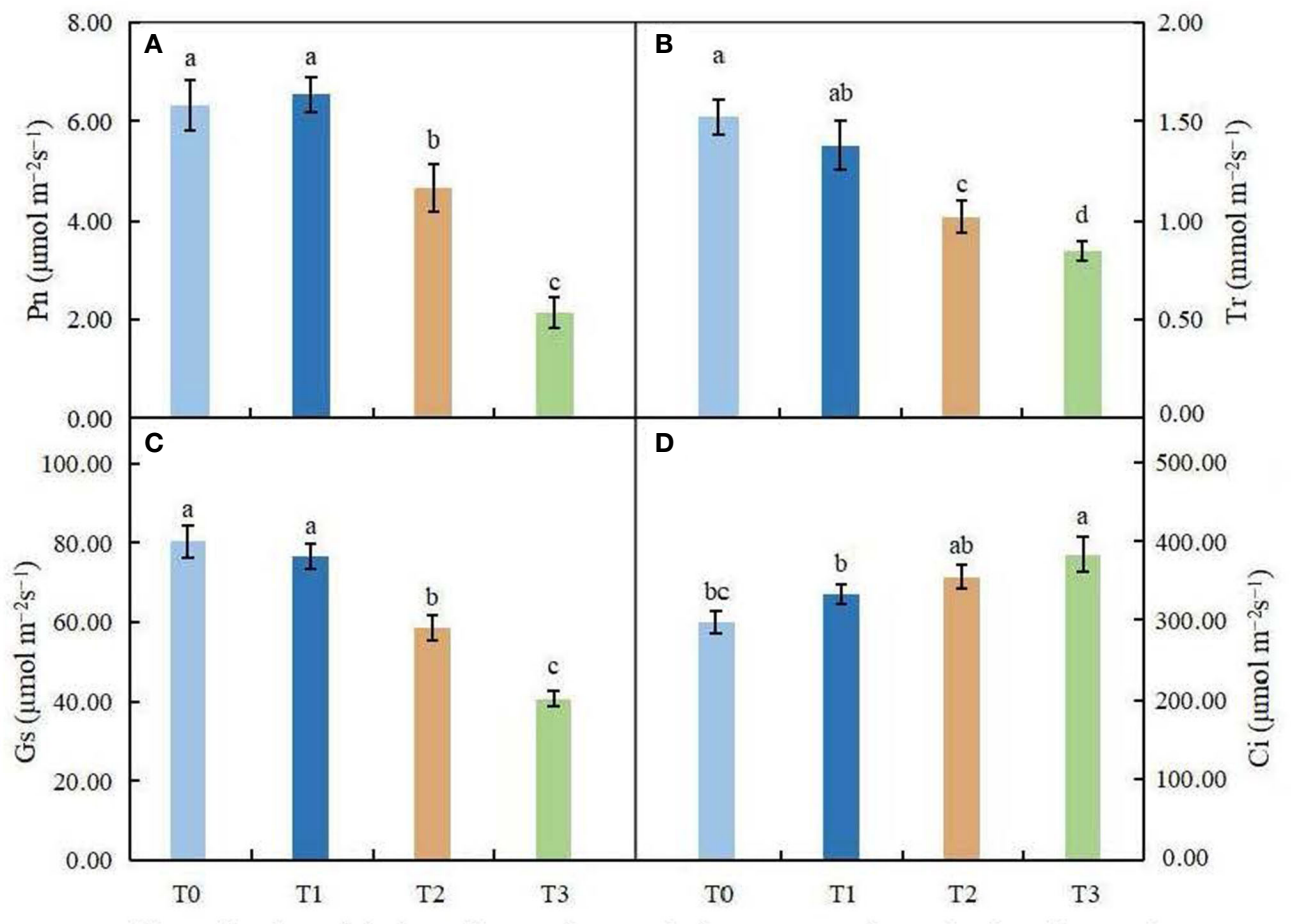
Effects of various light intensities on photosynthesis parameters in *C. betulus* after 60 days. *Pn*, net photosynthetic rate **(A)**; *Tr*, transpiration rate **(B)**; *Gs*, stomatal conductance **(C)**; *Ci*, intercellular CO_2_ concentration **(D)**. Different lowercase letters indicate significant differences between treatments (*P* < 0.05), n = 3.

The stomatal size, the palisade and parenchyma tissues in the leaves of *C. betulus* seedlings had different degrees of change under various levels of light intensity. The stomatal density and degree of stomatal opening decreased with decreasing light intensity, and the stomata were nearly closed in the T3 treatment (**Figures 2A, B**). **Figure 3C** shows a typical scanning electron micrograph of the cross-section of *C. betulus* leaves under the different shading treatments, including the upper and lower epidermis and palisade and spongy tissues. The palisade tissue of *C. betulus* was elongated and arranged in an orderly manner in the control (**Figure 3C**, T0). However, in the T1~T3 treatments, the leaf thickness decreased, the palisade and spongy parenchyma tissues gradually became loosely arranged, and the spaces between these tissues became larger.

### Effects of light intensity on photosynthetic parameters and chlorophyll fluorescence parameters

As shown in **Figures 3A–D**, Pn first increased and then decreased with increasing shading. There was no significant difference between T1 and CK of Pn, while under the T2 and T3 treatments, Pn decreased significantly (*P* < 0.05). The changes in Gs and Tr were consistent and decreased with increasing shading degree. In the treatment with minimal shading (T1), there was no significant difference between the two groups, but under the T3 treatment, Gs and Tr decreased by 44.08% and 49.12%, respectively, compared with the control. However, Ci increased with increasing shading degree, and the Ci of the T3 treatment group was significantly higher than that of the control group (*P* < 0.05). In conclusion, the treatment with minimal shading (T1) had little effect on the photosynthetic capacity of *C. betulus* seedlings, but under the moderate and severe shading treatments (T2 and T3), the photosynthetic capacity of *C. betulus* seedlings was adversely affected.

**Figure 4:**
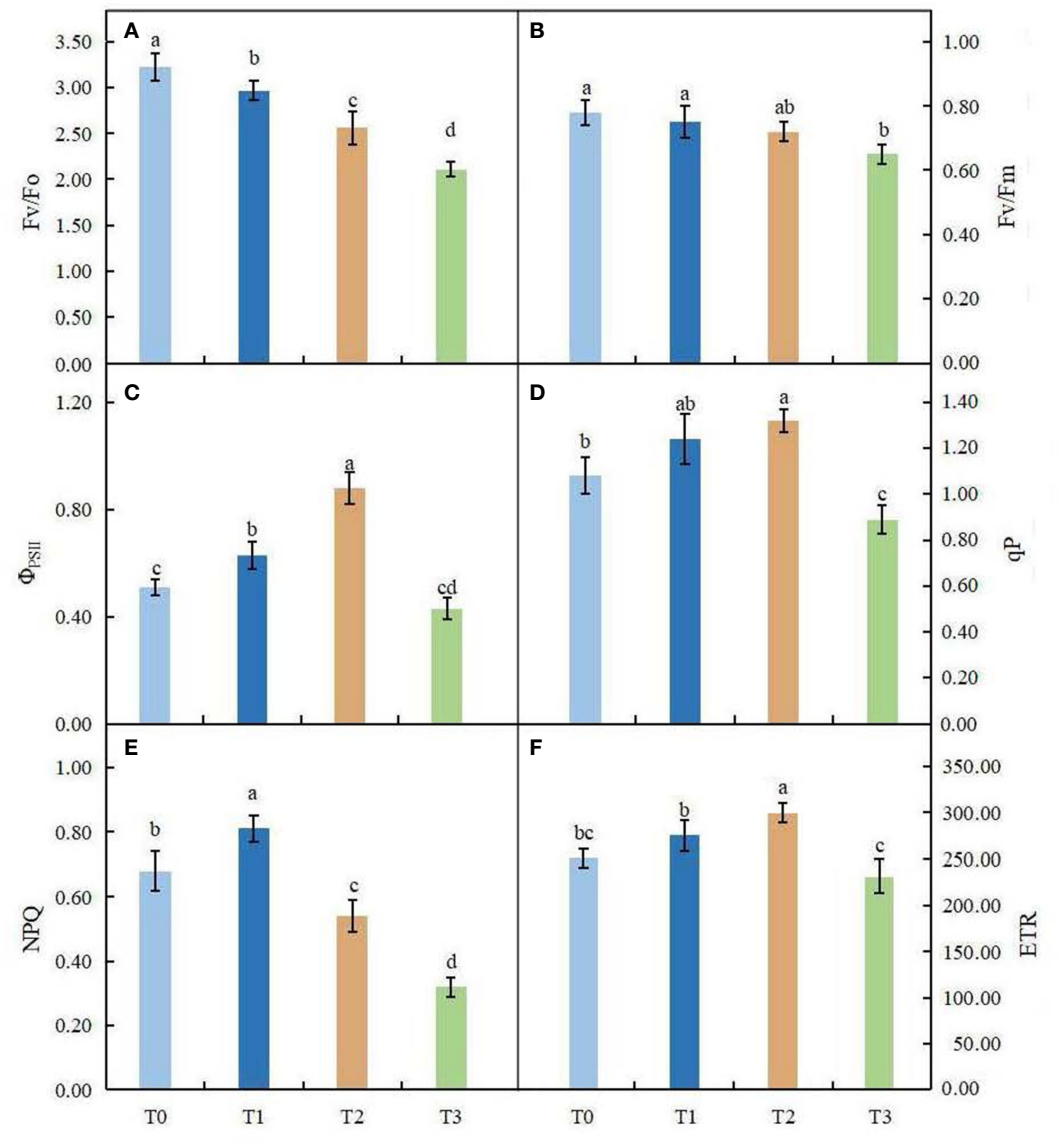
Effects of various light intensity on chlorophyll fluorescence parameters in *C. betulus* after 60 days. Fv/Fo, potential activity of PSII **(A)**; *Fv/Fm*, maximum quantum yield of photosystem II **(B)**;*Φ_PSII_*, photochemical quantum efficiency **(C)**; qP, photochemical quenching parameter **(D)**; NPQ, nonphotochemical quenching parameter **(E)**; ETR, electron transfer rate **(F)**. Different lowercase letters indicate significant differences between treatments (*P* < 0.05), n = 3.

Light intensity had different degrees of influence on the chlorophyll fluorescence parameters of *C. betulus* leaves (**Figures 4A–F**). The potential activity of PSII (Fv/Fo) and the maximum photochemical quantum yield of PSII (Fv/Fm) showed similar trends, decreasing with increasing shading degree, and were significantly different from the control under the severe shading treatment (T3). However, the changes in the actual photochemical quantum yield of PSII (Φ_PSII_), photochemical quenching coefficient (qP), nonphotochemical quenching coefficient (NPQ) and electron transport rate (ETR) were different from those of other indexes, which first increased and then decreased with increasing shading degree. Among these parameters, Φ_PSII_, qP and ETR were significantly higher in the T2 treatment than in the control, and NPQ was significantly higher in the T1 treatment than in the control (*P* < 0.05). In the T3 treatment, qP and NPQ were 17.59% and 52.94% lower, respectively, than in the control.

### Effects of light intensity on photosynthetic pigment contents

As shown in **Table 3**, shading had a significant impact on the contents of photosynthetic pigments in the leaves of *C. betulus.* The contents of chlorophyll a, b and total chlorophyll increased significantly compared with the CK (P < 0.05), as the degree of shading increased. The total chlorophyll content in the T1, T2 and T3 treatments was 1.22, 1.41 and 1.91 times that in the control (T0), respectively. These results indicated that the light capture ability of *C. betulus* seedlings under low light could be improved by increasing the chlorophyll content, thus improving the utilization efficiency of light energy. In contrast, the chlorophyll a/b value decreased with increasing shading degree, and the difference between each shade treatment and the control reached a significant level (*P* < 0.05), indicating that the increase in chlorophyll b content was greater than that in chlorophyll a content. The carotenoid content also increased as the degree of shading increased, and the difference with T0 was significant (*P* < 0.05).

**Table 3:** The main effects of light intensity on chlorophyll a content, chlorophyll b content, total chlorophyll content, chlorophyll a/b and carotenoid content of *C. betulus*.

Treatment	Chl a content (mg/g)	Chl b content (mg/g)	Total chlorophyll content (mg/g)	Chlorophyll a/b	Car content (mg/g)
**T0**	2.52 ± 0.18c	0.61 ± 0.04c	3.13 ± 0.32d	4.13 ± 0.68a	0.65 ± 0.06c
**T1**	3.04 ± 0.11b	0.78 ± 0.03b	3.82 ± 0.25c	3.89 ± 0.56b	0.98 ± 0.08b
**T2**	3.38 ± 0.22b	1.02 ± 0.05ab	4.40 ± 0.84b	3.31 ± 0.72c	1.25 ± 0.15ab
**T3**	4.54 ± 0.34a	1.25 ± 0.12a	5.99 ± 0.96a	3.13 ± 0.34c	1.48 ± 0.12a

The data in the table are the means ± SDs (n = 3); different lowercase letters in each column indicate significant differences between treatments (P < 0.05).

### Effects of light intensity on lipid peroxidation

**Figure 5:**
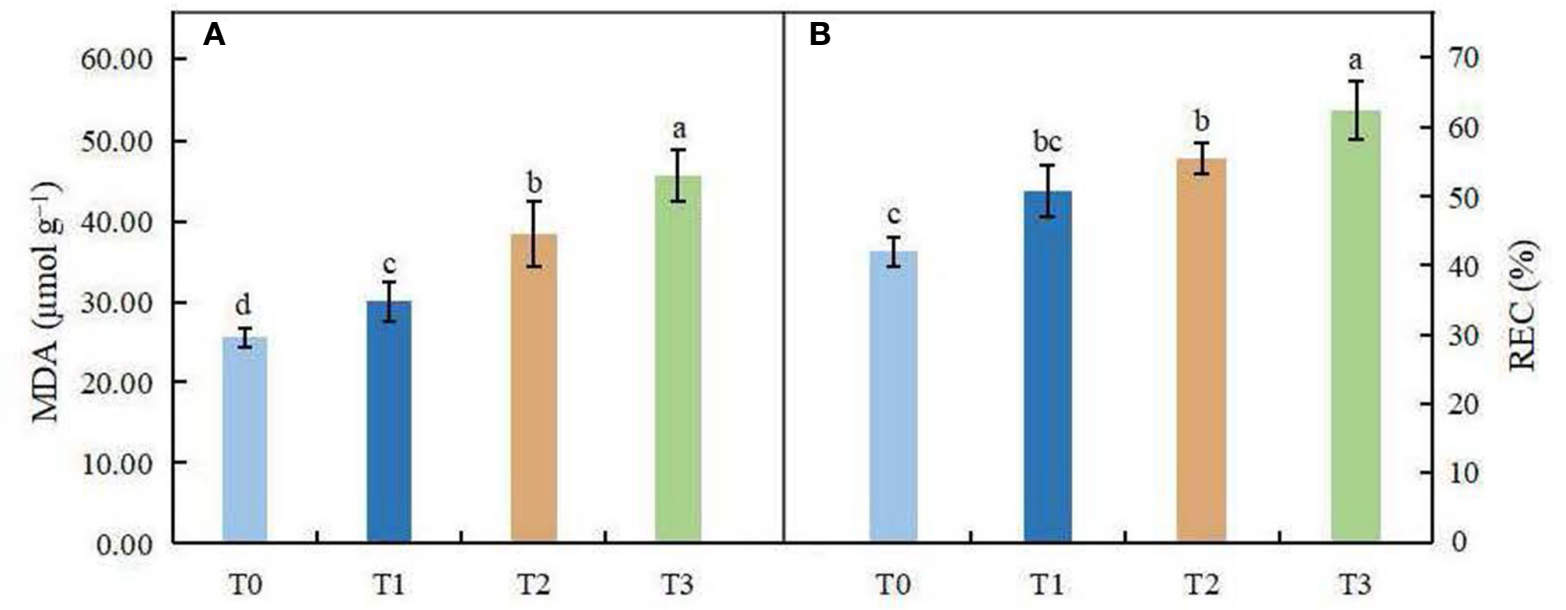
Effects of various light intensity levels on MDA content **(A)** and relative electrolytic conductivity (REC, **B**) in *C. betulus* after 60 days. Different lowercase letters indicate significant differences between treatments (*P* < 0.05), n = 3.

The changes in MDA content and relative electrical conductivity (REC) in leaves of *C. betulus* are shown in **Figures 5A, B**. With the decrease in light intensity, MDA content and REC showed a gradually increasing trend. ANOVA showed that MDA content in leaves was significantly different among different treatments (*P* < 0.05), indicating that shading affected MDA accumulation in leaves of *C. betulus*. The REC under the T1 treatment was not significantly different from that under the control, but in the T2 and T3 treatments, it was significantly higher than that of the control (*P* < 0.05).

### Effects of light intensity on organic osmolytes

**Figure 6:**
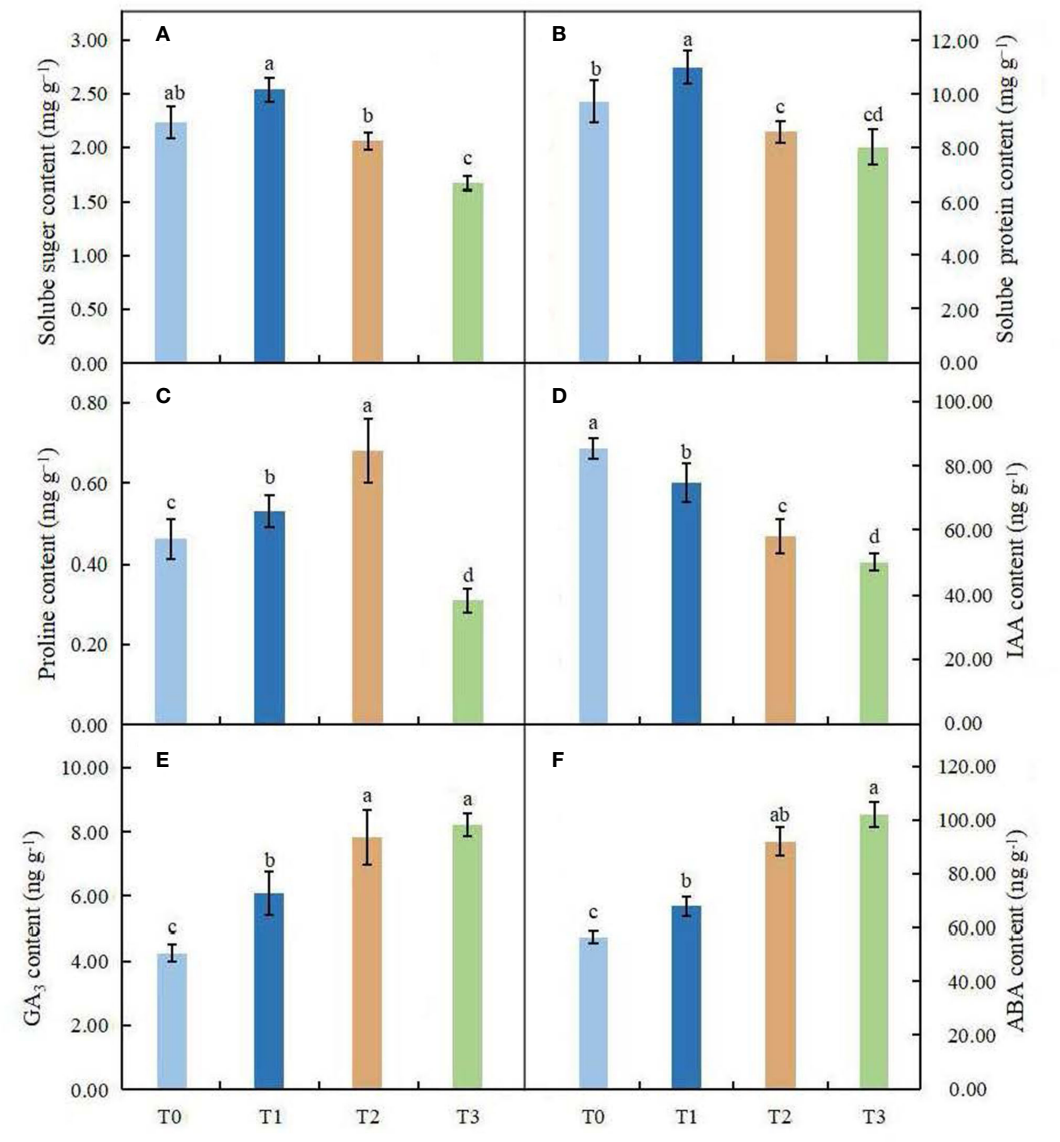
Effects of various light intensities on the soluble sugar content **(A)**, soluble protein content **(B)**, proline content **(C)**, IAA content **(D)**, GA_3_ content **(E)** and ABA content **(F)** in leaves of *C. betulus* after 60 days. Different lowercase letters indicate significant differences between treatments (*P * < 0.05), n = 3.

The soluble sugar content did not change significantly in leaves under the T1 and T2 treatments, but in the high shading treatment (T3), it was significantly lower than that in the control (**Figure 6A**). Compared with the control, the soluble protein content was significantly affected by different shading treatments (*P* < 0.05). In the T1 treatment, the content of soluble proteins was higher than that in the control, and a great reduction in protein content was found in the T2 and T3 treatments (**Figure 6B**). We observed that the proline content increased obviously in leaves under the T1~T2 treatments; however, the proline content was significantly lower in the high shading treatment (T3) than in the control group (**Figure 6C**).

### Effects of light intensity on endogenous hormones

The contents of endogenous hormones in leaves of *C. betulus* under different light treatments were significantly different from those in the control (*P* < 0.05, **Figures 6D–F**). The content of IAA decreased with decreasing light intensity and reached the minimum value (50.52 ng·g^-1^) in the T3 treatment, with a value that was 41.16% lower than that in the control. However, the contents of GA_3_ and ABA increased gradually with decreasing light intensity under different light conditions and reached their maximum values in the T3 treatment.

### Effects of light intensity on antioxidant enzyme activities

**Figure 7:**
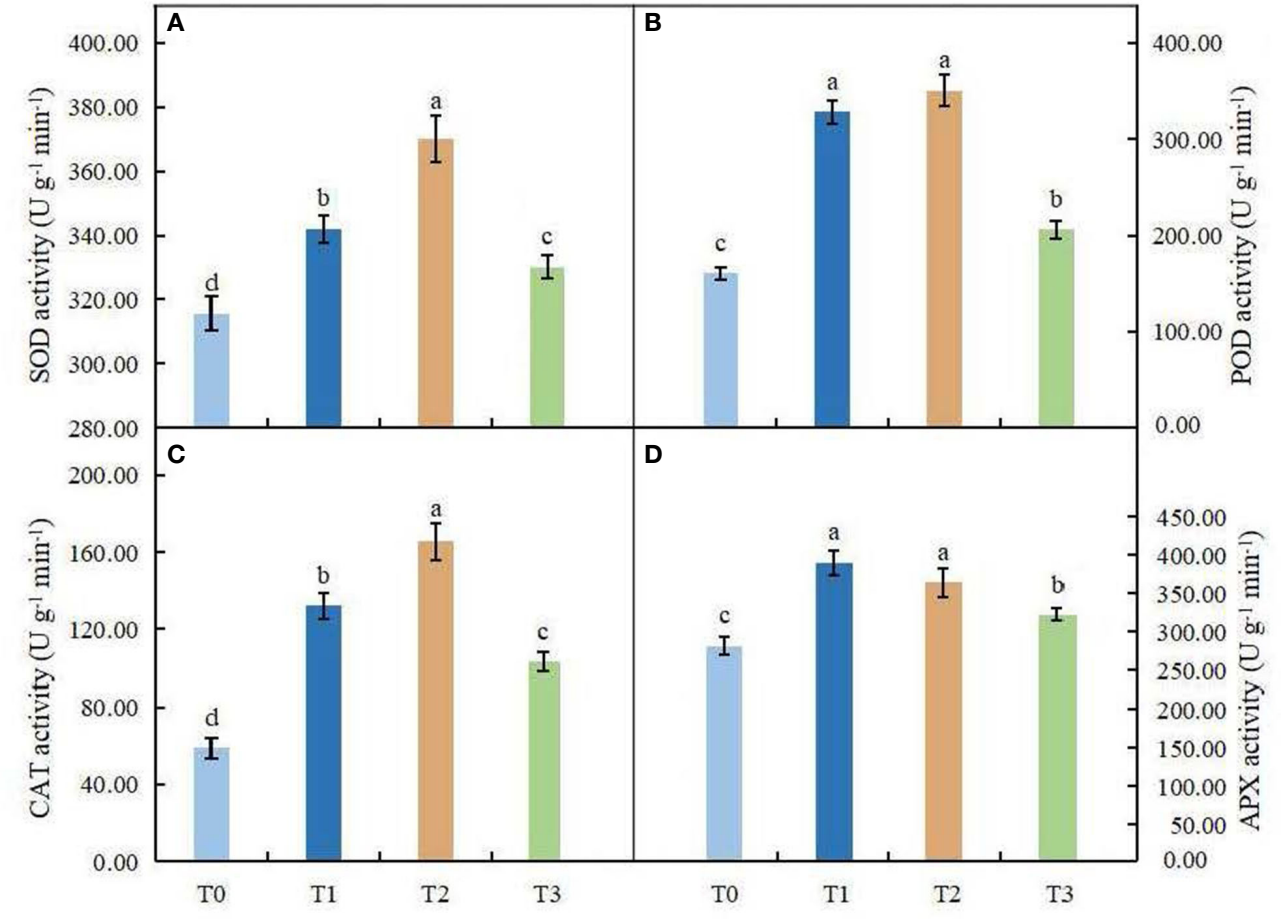
Effects of various light intensities on SOD activity **(A)**, POD activity **(B)**, CAT activity **(C)**, and APX activity **(D)** in leaves of *C. betulus* after 60 days. Different lowercase letters indicate significant differences between treatments (*P* < 0.05), n = 3.

The effect of light intensity on antioxidant enzyme activities in leaves of *C. betulus* is shown in **Figures 7A-D**. ANOVA results indicated that the antioxidant enzyme activities were significantly different between the different treatments and the control (*P* < 0.05). The SOD, POD and CAT activities of *C. betulus* leaves increased in the T1 and T2 treatments, reached a maximum value in the T2 treatment, and then decreased in the T3 treatment. The shading treatments caused a significant increase in the activity of APX after 60 days, and the maximum value (388.58 U·g^-1^ min^-1^) was found in the T1 treatment. Although the activity of APX decreased under the T2 and T3 treatments, it was still significantly higher than that in the control, by 29.37% and 14.41%, respectively.

## Discussion

Light intensity is an important environmental factor that is closely related to plant growth and development. When the external light environment changes, the growth, biomass and material distribution of plants will be affected to a certain extent. Under normal circumstances, the biomass and relative growth rate of plants will become lower in low-light environments (Pik et al., 2020). To adapt to shaded environments, plants usually undergo growth and morphological adjustments, a process called morphological plasticity, to adapt to the complex light environment. Some studies have shown that when plants are exposed to shade stress, they tend to have a decreasing base diameter and increasing plant height; that is, they show the characteristics of being ‘slender’. This may be because to obtain the maximum amount of light, the plant reduces the carbon used for basal diameter growth and allocates more assimilated carbon to vertical plant growth to capture more light energy (Liu C. et al., 2018). It was found in this study that with the decrease in light intensity, the plant height growth increased, but the ground diameter increment, taproot length, root, stem, leaf and total biomass decreased continuously. These results indicated that shading had a great effect on the growth of *C. betulus*, and the lower the light intensity was, the more restricted the growth. Biomass allocation patterns and their changes in aboveground and belowground parts are important representations of tree adaptation strategies to the environment (Guenni et al., 2018). Usually, when restricted by light conditions, trees allocate more resources to aboveground plant parts (Xue et al., 2022). By reducing the allocation of resources to root biomass and increasing the allocation of resources to leaf and stem biomass, trees will obtain more light resources, which is conducive to improving their ability to compete for light and therefore survive (Ma et al., 2014). The study of Toledo-Aceves and Swaine (2007) showed that with the weakening of light intensity, the allocation ratio of the root biomass of pioneer tree species in tropical rainforests decreased, while the allocation ratio of aboveground biomass increased. In this study, we found that the root:shoot ratio of *C. betulus* decreased with decreasing light intensity, which supported the above view. This finding indicated that the biomass allocation of the underground plant parts gradually became lower than that of the aboveground parts with the weakening of light to improve the ability of the plant to capture light and maintain plant growth.

Since leaves are the largest organ by which plants interact with the external environment, their morphological structure is very sensitive to changes in the ecological environment; therefore, changes in morphological and physiological characteristics can best reflect the influence of the light environment on plants and the ability of plants to adapt to the light environment (Fan et al., 2013; Yao et al., 2017). Studies have shown that most plants in a weak light environment can change blade shape, such as by increasing leaf length, leaf width, leaf area, leaf number and plant height to adapt to an environment with insufficient light intensity; in bright light, plants will adapt by increasing blade thickness, secreting a waxy layer and increasing leaf area to cope with strong light inhibition (Mokany et al., 2006; Klavins and Klavins, 2020). This study found that the leaf area and SLA of *C. betulus* increased with decreasing light intensity. This result showed that when the outside light intensity weakened, in order to capture more limited energy, plant height increased and the leaf area enlarged, thus reducing the consumption of plant nutrient substances by increasing the SLA, reducing the leaf thickness within a limited scope, and expanding the leaf area. These changes allowed the plant to more efficiently capture light energy and lower the compensation to the decline in the photosynthetic rate of the photon flux density phenomenon, thus enabling survival in the weak light environment, which is consistent with the view of Moreira et al. (2009). Leaves are the main site of photosynthesis, and changes in the anatomical characteristics of leaves will inevitably affect the photosynthetic efficiency of plants (Jumrani and Bhatia, 2020; Knauer et al., 2020). It was found that the thinness of the upper and lower epidermis and palisade tissues, the presence of palisade tissue cells with a low aspect:axis ratio, and the loose arrangement of the spongy tissue cells could improve light transmission and chloroplast light capture ability (Barbosa et al., 2021). This conclusion was further confirmed by the anatomical observation of leaves of *C. betulus* under different light intensities; that is, with the decrease in light intensity, the leaf thickness and presence of palisade tissue and spongy tissue of *C. betulus* decreased significantly, and the arrangement of cells in the spongy tissue gradually loosened, gradually increasing the gap between cells. This finding suggested that plants can adapt to different light environments by adjusting their leaf structures. The plasticity of plant leaf anatomical characteristics in response to changes in the light environment has been widely studied and has been confirmed in *Corylus avellana* (Catoni et al., 2015), *Sesleria nitida* (Puglielli et al., 2015) and other plants, and the patterns are basically consistent across species.

Photosynthesis by plants is a physiological process that is sensitive to environmental factors, and light intensity is one of the main factors affecting photosynthesis. Chlorophyll content, chlorophyll fluorescence parameters and photosynthetic parameters are important indexes for measuring the strength of plant photosynthesis (Dai et al., 2009; Zhang et al., 2017). Although different plants have different characteristics in response to changes in the light environment, under adversity and stress, plants will develop in the direction that is conducive to photosynthesis (Jutamanee and Rungwattana, 2017). In general, the decrease in the photosynthetic rate among plants under stress is mainly caused by stomatal limitation and nonstomatal limitation (Rezai et al., 2018). Stomatal limitation is due to the decrease in stomatal conductance, and CO_2_ from the external environment is restricted from entering the plant through the leaf stomata, which leads to a decrease in the photosynthetic rate. However, the reduction in the photosynthetic rate caused by nonstomatal limitation is mainly caused by the reduction in photosynthesis-related enzyme activities and the damage to photosynthetic organs caused by stress, and intercellular carbon dioxide may still be maintained at a high level at this time. Ci is an important basis for judging whether stomatal factors cause changes in the photosynthetic rate (Takeda and Maruta, 2008). Ci is positively correlated with Pn, Tr and Gs, indicating that the decline in photosynthesis is caused by stomatal factors hindering CO_2_ utilization; otherwise, it is caused by nonstomatal factors. The results of this study showed that with the decrease in shading, the trend of Pn in *C. betulus* was basically consistent with that of Gs and Tr, which showed overall decreasing trends, and the lowest value was reached at 25% light intensity. However, the content of Ci was negatively correlated with the Pn, Tr and Gs, which indicated that photosynthesis in *C. betulus* was affected by nonstomatal factors, and the decrease in the leaf photosynthetic rate was caused by the restriction of Rubp carboxylation and photochemistry. A similar phenomenon has been observed in *Asarum forbesii* (Lu et al., 2022) and *Emmenopterys henryi* (Li et al., 2020). This further indicated that when the light intensity was too low, the plants had a light deficit and could not obtain the necessary light energy for life-sustaining activities, resulting in the lowest photosynthetic rate.

Chlorophyll fluorescence, part of the photosynthetic process, mainly reflects the efficiency of light absorption, transmission and utilization by plants and is commonly used to characterize the level of photosynthetic efficiency of plants (Genty et al., 1989; Pashkovskiy et al., 2018). Fv/Fm is an indicator of the photoconversion efficiency of PSII, while Fv/Fo indicates the potential activity of the PSII photoreaction center. The higher the Fv/Fm and Fv/Fo are, the higher the photoconversion efficiency of PSII (Zhang, 1999). Baker (2013) found that the Fv/Fm of plants is generally 0.75~0.85 under normal conditions. In this experiment, the Fv/Fm and Fv/Fo in the treatment groups were lower than those in the control group with the increase of shading degree (*P* < 0.05), indicating that the open proportion of the PSII reaction center in *C. betulus* under the shading treatment decreased and the light energy utilization efficiency was reduced. NPQ reflects nonphotochemically dissipated energy, which is one of the photoprotective mechanisms of plants (Heraud and Beardall, 2000). In this experiment, Φ_PSII_, qP, ETR and NPQ of *C. betulus* leaves increased first and then decreased with increasing shading degree, and under the severe shading treatment (T3), Φ_PSII_, qP, ETR and NPQ were significantly lower than those under the other shading treatment groups, indicating that the photosynthetic apparatus of *C. betulus* leaves may be partially inactivated or damaged under severe shading conditions. The primary photochemical activity of PSII decreased, the photosynthetic electron transport rate was inhibited, the proportion of heat energy dissipation in the absorbed light energy increased, and the utilization efficiency of absorbed light energy of PSII decreased, resulting in a decrease in the photosynthetic rate. These results indicated that *C. betulus* had a certain adaptability to low light, but this ability was limited. Light intensity that is too low reduces the photosynthetic efficiency of plants and affects the normal growth of plants.

Chlorophyll has the function of absorbing and transferring light photons and is an important component in the maintenance of normal photosynthesis of plants. Its content is closely related to the site conditions of plant growth and the characteristics of plants (Agathokleous et al., 2020). Chl a and Chl b are the main photosynthetic pigments, and carotenoids can absorb light energy and transfer it to chlorophyll to assist plants in photosynthesis (Liu et al., 2014). The content and proportion of these pigments are important indicators for plants to cope with environmental stress (Chai et al., 2018). In this study, the contents of Chl a, Chl b, Chl (a+b) and carotenoids in *C. betulus* increased gradually compared with the CK with decreasing light intensity, which indicated that low light intensity may beneficial to the synthesis of chlorophyll and carotenoid, and made the treatment groups show greener leaf color than the control (**Figure 1**). *C. betulus* seedlings could improve the efficiency with which it captured light radiation by increasing the chlorophyll content in low-light environments, thus adapting to different low-light environments. The chlorophyll a/b value is one of the important indexes to measure the shade tolerance of plants, and plants can adapt to shading by reducing chlorophyll a/b (Dai et al., 2009). Some studies have suggested that the chlorophyll a/b value of shade plants is below 3.0 (Lichtenthaler et al., 1981; Hoflacher and Bauer, 1982). The Chl a/b of *C. betulus* was positively correlated with light intensity, decreasing with the decrease in light intensity, and the Chl a/b values were all higher than 3.0, indicating that *C. betulus* is a typical plant with high requirements for light intensity in the growing environment.

Osmotic regulatory substances play an important role in plant resistance to abiotic stress. When reactive oxygen species are produced rather than antioxidants, the cytoplasmic membrane can perform substantial membrane lipid peroxidation; at this time, the permeability of the plant cell membrane will increase, leading to cytoplasmic extravasation and physiological and metabolic plant disorders (Ramanjulu and Bartels, 2002). Then, the plants will reduce the cell water loss caused by stress by adjusting the contents of osmotic regulatory substances such as SS, SP and Pro to maintain the balance of cell osmotic pressure and thus reduce the degree of damage caused by stress (Hamouda et al., 2015). SS and SP are not only important nutrients but also participate in the osmoregulation process of plant cells, and SS can also remove ROS in vacuoles and chloroplasts and indirectly activate the antioxidant enzyme system (Van and Valluru, 2009). Pro has been shown to be an effective free radical scavenger that plays an important role in stabilizing protoplasmic colloids and cell metabolic processes and maintaining osmoregulatory balance (Mutava et al., 2015). This study found that the contents of SS and SP in *C. betulus* leaves first increased and then decreased with decreasing light intensity, suggesting that under mild shade stress, *C. betulus* can remove ROS from vacuoles and chloroplasts by increasing SS and SP contents and maintain turgor pressure by osmotic regulation through the synergistic effect of carbon and nitrogen metabolism pathways. However, under moderate and severe shading stress, the SS and SP contents gradually decreased to their lowest levels in the T3 treatment, which may be due to the lack of light. The rate of photosynthesis in the leaves decreased sharply, and the synthesis of SS and SP was blocked; these effects will lead to a shortage of nutrients in plants, causing adverse effects on growth. The Pro content in *C. betulus* increased under mild and moderate levels of shade (i.e., the T1 and T2 treatments) but decreased significantly under heavy shade conditions (the T3 treatment), suggesting that the Pro content of *C. betulus* can still be maintained at a relatively high content under appropriate shading conditions, scavenging free radicals in plant cells and counteracting the effect of light intensity factors on cell osmoregulation, while its synthesis is blocked in extremely low light, thus limiting the effect of osmoregulation.

ROS are one-electron reduction products of a class of oxygen, which are important signal transduction substances in plants (Kaushik and Aryadeep, 2014). A study found that when plants suffer adversity stress, on the one hand, ROS material will stimulate plants to produce a series of physiological and biochemical reactions and resistance to environmental stresses of the outside world; on the other hand, when the generation and removal of ROS in plant cells are in an unbalanced state, membrane lipid peroxidation will occur in the cell membrane, and protein oxidation will cause damage to the cell membrane, resulting in the imbalance of normal physiological metabolism of plants and even leading to the death of plants (Pareek et al., 2017). MDA is the final product of membrane lipid peroxidation and one of the important signs of membrane system damage (Sofo et al., 2004). This study found that the MDA content and relative conductivity of *C. betulus* leaves increased with decreasing light intensity, which was the lowest under natural light intensity and the highest under the T3 condition. These results indicated that shading caused low-light stress in *C. betulus*, which led to damage to membrane function or membrane structure, an increase in membrane permeability, and serious membrane lipid peroxidation, and the same phenomenon was also found in *Quercus wutaishanica* (Zhang et al., 2021). SOD, POD and CAT are important antioxidant enzymes (Zhao et al., 2019). When plants are under environmental stress, ROS can induce their activity to increase and remove the accumulated O^·-2^ and H_2_O_2_ in the cells, thus playing a protective role for plant cells (Koca et al., 2006). SOD first converts O^·-2^ into H_2_O_2_ and O_2_ and then dissociates the generated H_2_O_2_ and endogenous H_2_O_2_ to stimulate POD and CAT activities, and the three synergistic processes eliminate excessive reactive oxygen species in plants (Neto et al., 2006). Studies have found that shading, drought, salinity and other stresses can induce the activities of SOD, POD, CAT and other antioxidant enzymes and increase the ability of plants to remove reactive oxygen species, but this process is species-specific and varies with different stress types (Favaretto et al., 2011; Bharuth et al., 2020; Liu et al., 2022). This study found that the activities of SOD, POD, CAT and APX in leaves of *C. betulus* first increased and then decreased with increasing shading intensity, but the activities of protective enzymes were higher than those of the control under different shading conditions. This indicated that under a low-light environment, *C. betulus* can regulate the activity of protective enzymes in the body to remove the O^·-2^ and H_2_O_2_ produced by shading stress. However, under severe shading conditions, the metabolic balance in plants was disrupted, and the enzyme activity and free radical scavenging function were irreversibly decreased, resulting in severe membrane lipid peroxidation in *C. betulus*. With the decrease in light intensity, the protective enzyme activities of *C. betulus* showed the same trend, indicating that there was an obvious synergistic effect of antioxidant enzymes *in vivo*.

Auxin (IAA), gibberellin (GA_3_) and abscisic acid (ABA) are important endogenous hormones in plants. Studies have shown that light is not only an important environmental factor for plant photosynthesis but can also regulate plant growth and development through endogenous hormones by using genes related to photoreceptor signaling (Jiang et al., 2021). Cui et al. (2014) found that shading decreased the contents of IAA and GA_3_ and increased the contents of ABA in the leaves of summer maize. In this study, the content of IAA decreased with decreasing light intensity, but the contents of GA_3_ and ABA showed the opposite trend, which differed from the above research results. ABA mainly inhibits cell division and elongation and inhibits the growth of roots, stems, leaves and other organs (Khorshidi and Nojavan, 2006). The ABA content in leaves of *C. betulus* increased under shaded conditions, which strengthened the inhibitory effect of shading on leaf growth. GA_3_ can promote leaf growth and maintain leaf function, and an increase in GA_3_ content can promote the growth of leaves (Ulger et al., 2004). GA_3_ would also promote the seedling height, the increase of plant height increment in low light conditions may be due to the increase of GA_3_ content. What’s more, studies have also shown that GA_3_ in leaves can inhibit chlorophyll decomposition, and the two have a synergistic effect (Li et al., 2010), that may be the reason of why chlorophyll content in *C. betulus* increased as the degree of shading increased. Therefore, on the one hand, low light intensity increased GA_3_ and maintained leaf development. On the other hand, reducing the IAA content and increasing the ABA content resulted in leaf thinning and reduced dry matter accumulation. Therefore, the synergistic effect of different endogenous hormones promoted the elongation and broadening of leaves and increased the leaf area of *C. betulus* under low light conditions.

**Figure 8:**
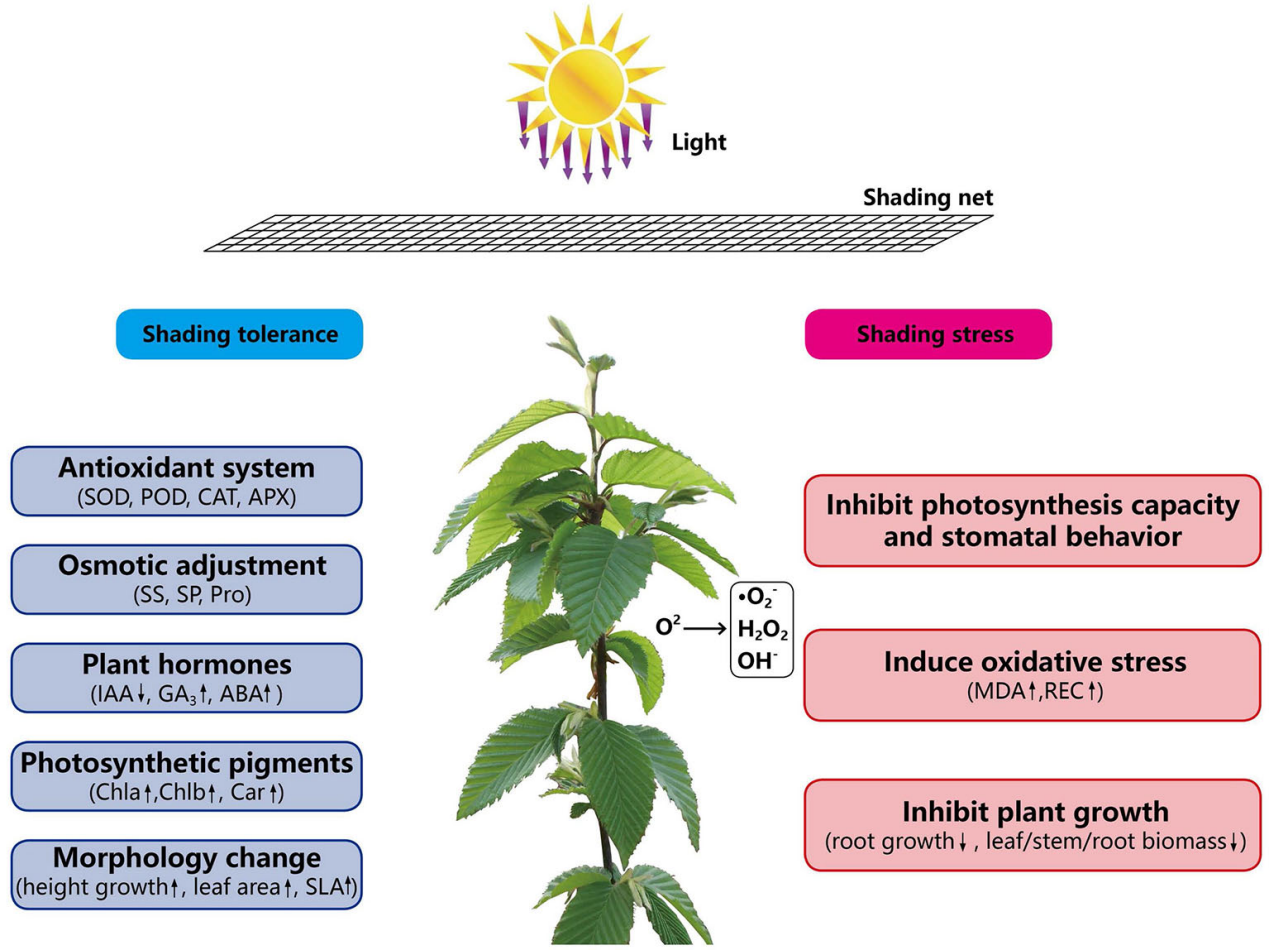
The mechanism of shading response of *C. betulus* seedlings.

Shade tolerance is determined by genetic characteristics of plants and many biological and abiotic factors. In this study, the morphological, physiological and ecological response of *C. betulus* seedlings under different light intensities were studied (**Figure 8**). In the future, further studies can be conducted from the perspective of proteins and molecules levels to comprehensively analyze the mechanism of shade tolerance in plants.

## Conclusions

This study presents new information on the growth and ecophysiological responses of *C. betulus* seedlings to different light intensities. *C. betulus* was affected by shading stress through the inhibition of plant biomass accumulation, photosynthetic capacity and stomatal behavior, and increas ing MDA content and relative electrolytic conductivity, suggesting a limited degree of shade tolerance. Our results found that *C. betulus* seedlings can activate relevant mechanisms to adapt to low light stress, such as adjusting growth and leaf morphology, accumulating relatively high concentrations of organic solutes to maintain cellular osmotic balance and improving the activity of antioxidant enzymes as a defense against shading stress. A reduction in the growth of *C. betulus* seedlings was observed under severe shading conditions (T3). *C. betulus* seedlings can adapt to sunlight, grow best in natural light, and can tolerate certain degrees of shading, which is capable of growing under shading with 75% or 50% light transmittance. However, under heavy shading (25% light transmittance), *C. betulus* develops insufficient resistance to low-light environments and has a difficult time growing. Our study provides a better understanding of the responses of *C. betulus* to low-light stress at the physiological and biochemical levels. This ornamental tree species has the potential to be cultivated in the middle and upper layers of landscape gardens in China.

## Data availability statement

The original contributions presented in the study are included in the article/supplementary material. Further inquiries can be directed to the corresponding author.

## Author contributions

QZ and ZZ conceived and designed the experiments; QZ, FZ, and HZ performed the experiments and analyzed the data; and QZ wrote the paper. All authors read and approved the final paper.

## Funding

This research was funded by the National Natural Science Foundation of China (31770752), the Jiangsu Science and Technology Support Program (BY2015006–01), the 333 Projects of Jiangsu Province (BRA2018065), and the Natural Science Foundation for Universities of Jiangsu Province (20KJB220006) and is a project of the 13th Five-Year Plan of Jiangsu Open University (2020-D-01).

## Acknowledgments

We would like to thank Jing Yang, a laboratory specialist at the Advanced Analysis Testing Center (AATC), Nanjing Forestry University, China, for assistance with the observations of the microstructures of the plants. We express our gratitude to Feibing Wang, a research assistant at the College of Landscape Architecture, Nanjing Forestry University, China, for her help cultivating the seedlings.

## Conflict of interest

The authors declare that the research was conducted in the absence of any commercial or financial relationships that could be construed as a potential conflict of interest.

The reviewer YX declared a shared affiliation with the author HZ, ZZ to the handling editor at the time of review.

## Publisher’s note

All claims expressed in this article are solely those of the authors and do not necessarily represent those of their affiliated organizations, or those of the publisher, the editors and the reviewers. Any product that may be evaluated in this article, or claim that may be made by its manufacturer, is not guaranteed or endorsed by the publisher.
